# Prevalence of antimicrobial resistance in Somalia: A systematic review

**DOI:** 10.1016/j.ijregi.2025.100800

**Published:** 2025-11-04

**Authors:** Shafie Abdulkadir Hassan, Mohamed Hassan Osman, Mowlid Abdikarin Mohamed

**Affiliations:** 1 Center for Antimicrobial Resistance Research, Jamhuriya University of Science and Technology, Mogadishu, Somalia; 2 Department of Medical Laboratory Sciences, Faculty of Medicine and Health Sciences, Jamhuriya University of Science and Technology, Mogadishu, Somalia; 3 Laboratory Department, Royal Hospital, Mogadishu, Somalia

**Keywords:** Prevalence, Antimicrobial resistance, Systematic review, Somalia

## Abstract

**Objectives:**

Antimicrobial resistance (AMR) is a severe but poorly documented threat in Somalia due to its fragmented healthcare system. This study reviews AMR prevalence and clinical impact in Somalia, comparing the findings to regional trends to address this critical data gap.

**Methods:**

This study was a systematic literature review of antimicrobial susceptibility patterns in Somalia. Data on resistance rates for common Gram-positive and Gram-negative bacteria were extracted, calculated as a proportion of resistant isolates, and then narratively synthesized in summary tables.

**Results:**

The data reveal a critical state of AMR in Somalia. Among Gram-positive bacteria, there is a near-total prevalence of methicillin-resistant *Staphylococcus aureus* at 97.4%. Gram-negative bacteria like *Escherichia coli* show extensive resistance to common treatments (94.6% to trimethoprim-sulfamethoxazole). Multidrug resistance is rampant, with pathogens such as *Acinetobacter baumannii* exhibiting 100% resistance to multiple drug classes, including Reserve group carbapenems. These rates are markedly higher than in neighboring Ethiopia, highlighting a severe and escalating public health emergency.

**Conclusions:**

Somalia’s AMR crisis has rendered standard antibiotics ineffective. Urgent action is required, including national surveillance, local treatment guidelines, and antimicrobial stewardship to control this threat.

## Highlights

• Antimicrobial resistance in Somalia has reached catastrophic levels.
• Near-universal methicillin-resistant *Staphylococcus aureus* prevalence was found at 97.4%.
• Common urinary tract infection treatments are ineffective due to high *Escherichia coli* resistance.
• Extensively drug-resistant “superbugs” are now circulating.
• Standard empirical antibiotic therapies are dangerously unreliable.
• Urgent national surveillance and stewardship programs are essential.

## Introduction

Inappropriate use of antimicrobial agents contributes to the development and spread of antimicrobial resistance (AMR), a phenomenon that threatens to undermine modern medicine by rendering common infections untreatable [1,2]. The World Health Organization (WHO) has declared AMR one of the top 10 global public health threats facing humanity [3]. While AMR is a global crisis, its burden is not evenly distributed. Sub-Saharan Africa faces one of the world’s highest AMR-associated mortality rates, driven by a combination of a high burden of infectious diseases, weak health systems, poor sanitation, and the unregulated availability of antimicrobial drugs [4,5].

Recent analyses indicate that Africa bears one of the highest burdens of AMR globally, with an estimated 4.95 million deaths associated with bacterial AMR in 2019 [6]. The WHO African Region alone accounted for significant mortality due to resistant pathogens [7]. Alarmingly, a recent European Society of Clinical Microbiology and Infectious Diseases report estimated that over 3 million children died from AMR-related infections in 2022, largely driven by inappropriate use of Watch and Reserve antibiotics [8,9]. Beyond health consequences, AMR imposes major economic costs, with the World Bank estimating global Gross Domestic Product losses exceeding $1 trillion annually by 2050 if unchecked [10,11].

In response, the WHO launched the Global Action Plan on AMR in 2015, prompting many countries, including Somalia, to develop National Action Plans to contain resistance [12,13]. The WHO Global Antimicrobial Resistance and Use Surveillance System and the AWaRe (Access, Watch, Reserve) framework now guide rational antibiotic use and surveillance globally [14] Irrational antibiotic prescribing remains widespread in low- and middle-income countries (LMICs), largely driven by limited knowledge of AMR and weak antimicrobial stewardship (AMS) programs, as demonstrated by recent studies showing persistently high rates of inappropriate antibiotic use in primary care [15,16].

Somalia presents a unique and particularly alarming case [17,18]. Over three decades of civil conflict and political instability have led to a fragile and fragmented healthcare system, with limited governance and regulatory oversight [19,20]. This environment has created a “perfect storm” for the emergence and spread of AMR [21]. The unregulated sale of antibiotics, often without a prescription, is widespread [22,23]. Diagnostic capacity is severely limited, forcing clinicians to rely on empirical treatment, which is often inappropriate [24]. Furthermore, basic infection prevention and control measures are difficult to implement and sustain in many healthcare settings [25]. These factors have placed Somalia among the top 10 countries with the highest AMR-associated mortality rates globally [17].

While the scale of the problem is evidently massive, there has been a significant lack of synthesized, comprehensive data to guide clinical practice and public policy in Somalia. Although previous literature reviews on antimicrobial resistance in Somalia exist, no systematic review has been conducted [17]. This study is justified as it provides a structured and comprehensive synthesis of all available evidence, offering more reliable estimates of AMR prevalence, identifying data gaps, and supporting evidence-based decision-making.

Therefore, this study aims to systematically review the available literature to determine the prevalence of AMR among the most common bacterial pathogens in Somalia. By consolidating existing data, this review seeks to provide a clear overview of the AMR landscape, critically evaluate its clinical implications by comparing it with regional data from countries like Ethiopia, and propose evidence-based recommendations for policy and practice. This study also categorizes the antibiotics analyzed according to the WHO AWaRe classification to better contextualize patterns of use and stewardship needs.

## Methods

### Search strategy

A systematic literature search was conducted in PubMed, following the PRISMA 2020 guidelines, to identify all relevant studies on AMR in Somalia. Search terms included combinations of ``antimicrobial resistance,'' ``antibiotic resistance,'' ``drug susceptibility,'' ``Somalia,'' and the names of common bacterial pathogens. This review was registered in PROSPERO under registration number CRD4201132749.

### Eligibility criteria

Studies were included if they were original research articles, conducted in Somalia, involved human subjects, and reported quantitative data on the *in-vitro* susceptibility of bacterial isolates to one or more antimicrobial agents. Reviews, commentaries, case reports, and studies focusing on non-bacterial pathogens were excluded.

### Study selection and data extraction

**Figure 1:**
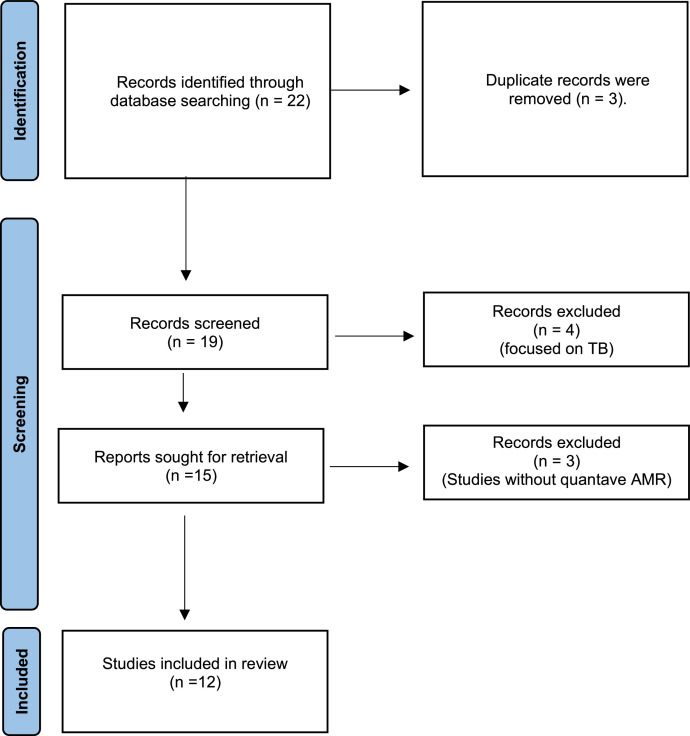
Flow of information through the different phases of a systematic review.

All identified articles were screened by title and abstract, followed by a full-text review of potentially eligible studies. For each included study, data were extracted onto a standardized form. Extracted data included the bacterial species, the number of isolates tested, the antimicrobial agents tested, and the number of isolates found to be resistant. As shown in the flow diagram, the systematic review began with 22 records identified through database searching. After removing three duplicates, 19 records were screened, of which four focusing on tuberculosis were excluded. Fifteen full-text reports were assessed, and three studies lacking quantitative AMR data were excluded. Ultimately, 12 studies were included in the final review (Fig. 1).

### Data synthesis and analysis

Due to the anticipated heterogeneity in study design and reporting, a narrative synthesis of the data was performed. The prevalence of resistance for each pathogen-antibiotic combination was calculated by dividing the total number of resistant isolates by the total number of isolates tested, with the result expressed as a percentage. The findings were aggregated and are presented in summary tables for Gram-positive and Gram-negative bacteria. The antibiotics were classified based on the WHO AWaRe system to facilitate international comparison and interpretation in the context of AMS.

## Results

### Study characteristics

This systematic review synthesized data from 12 studies across Somalia, which collectively analyzed 1531 bacterial isolates from a wide range of clinical conditions. Urinary tract infections were the most common focus, accounting for 728 isolates, with significant data also coming from otitis media and intra-abdominal infections.

### Antibiotic resistance profile of frequently isolated Gram-positive bacteria

The review identified extremely high levels of resistance among key Gram-positive pathogens, severely compromising the utility of essential “Access” group antibiotics (Table 1). *Staphylococcus aureus*, the most frequently reported species, exhibited a resistance profile indicative of widespread multi-drug resistance. Resistance to cefoxitin, a “Watch” group antibiotic, was 97.4%, suggesting a near-universal prevalence of methicillin-resistant *S. aureus* (MRSA) among the tested isolates (Table 2).

**Table 1:** Antimicrobial resistance profile of Access group antibiotics in Somalia.

Antibacterial agent	AWaRe category	*S. aureus* (%)	*S. pneumoniae* (%)	*S. agalactiae* (%)	Coagulase-negative staphylococci (%)	*E. coli* (%)	Klebsiella spp. (%)	*A. baumannii* (%)	*P. aeruginosa* (%)
Amoxicillin	Access	8.9	-	-	7.7	26	-	-	98.4
Amoxicillin-clavulanic acid	Access	29.4	-	-	-	58.9	-	100	57.9
Ampicillin	Access	90.9	100	-	48	73.5	100	-	94.1
Cefazolin	Access	-	-	-	-	82.9	84.6	-	92.9
Cephalexin	Access	-	-	-	-	46.2	-	-	93.5
Clindamycin	Access	73.5	-	-	25	38	-	-	-
Gentamicin	Access	32.3	60	-	42.3	32.3	25	-	47.4
Nitrofurantoin	Access	11.1	-	-	-	11.7	33.3	-	38.4
Penicillin	Access	35.1	-	100	33.3	57.9	-	-	78.1
Tetracycline	Access	37.1	-	-	-	73.3	40	-	33
Trimethoprim-sulfamethoxazole (Co-trimoxazole / SMX-TMP)	Access	61.1	-	-	-	94.6	50	100	62.3

**Table 2:** Antimicrobial resistance profile of Watch group antibiotics in Somalia.

Antibacterial agent	AWaRe category	*S. aureus* (%)	*S. pneumoniae* (%)	*S. agalactiae* (%)	Coagulase-negative staphylococci (%)	*E. coli* (%)	Klebsiella spp. (%)	*A. baumannii* (%)	*P. aeruginosa* (%)
Amikacin	Watch	-	-	-	-	5	11.1	63.4	17.4
Azithromycin	Watch	-	-	-	-	8.3	-	-	-
Cefotaxime	Watch	-	-	-	-	85.4	100	-	81.3
Cefoxitin	Watch	97.4	-	-	50	60.3	87.5	-	83.3
Ceftriaxone	Watch	55.5	-	-	-	61.7	87.5	100	50
Cefuroxime	Watch	-	-	-	-	83.4	79.7	100	40.1
Ciprofloxacin	Watch	84.2	-	-	21.7	53.3	33.3	97.8	87.4
Clarithromycin	Watch	38.1	30	-	28.2	38.1	33.3	-	16.7
Ertapenem	Watch	100	-	-	-	17.2	33.3	98.9	34.8
Erythromycin	Watch	27.1	30	-	25	-	-	-	62.2
Levofloxacin	Watch	72.7	-	-	-	39.9	11.1	100	31.3
Meropenem	Watch	22.2	-	-	-	12.6	-	100	20.5
Piperacillin-tazobactam	Watch	-	-	-	-	47.4	45.5	-	29.8
Vancomycin	Watch	-	-	-	-	5	-	-	55.5

Similarly, resistance was exceptionally high against crucial “Access” group agents like ampicillin (90.9%) and clindamycin (73.5%), rendering these common first-line treatments largely ineffective (Table 1). High resistance was also observed for ciprofloxacin (84.2%), a “Watch” group fluoroquinolone (Table 2). Encouragingly, resistance to the “Reserve” antibiotic linezolid (3.6%) was low, highlighting its critical importance as a last-resort option (Table 3). Gentamicin, an “Access” agent, showed a comparatively lower resistance rate of 32.3% (Table 1).

**Table 3:** Antimicrobial resistance profile of Reserve group antibiotics in Somalia.

Antibacterial agent	AWaRe category	*S. aureus* (%)	*S. pneumoniae* (%)	*S. agalactiae* (%)	Coagulase-negative staphylococci (%)	*E. coli* (%)	Klebsiella spp. (%)	*A. baumannii* (%)	*P. aeruginosa* (%)
Cefepime	Reserve	-	-	-	-	51.8	50	98.9	95.9
Ceftazidime	Reserve	11.1	-	-	-	45.8	81.8	98.9	19.6
Colistin	Reserve	-	-	-	-	4.5	-	2.3	73.7
Fosfomycin	Reserve	-	-	-	-	29	-	-	75.6
Linezolid	Reserve	3.6	-	-	-	13.1	-	-	-
Piperacillin	Reserve	-	-	-	-	26.25	-	100	65

Other Gram-positive pathogens showed equally concerning patterns. *Streptococcus pneumoniae* demonstrated 100% resistance to the “Access” antibiotic ampicillin (Table 1), while coagulase-negative staphylococci showed 50% resistance to the “Watch” group agent cefoxitin (Table 2) and the “Access” drug co-trimoxazole (Table 1).

### Antibiotic resistance profile of frequently isolated Gram-negative bacteria

Gram-negative pathogens demonstrated extensive resistance patterns that threaten even “Reserve” group antibiotics. Escherichia coli, a primary cause of urinary tract infections (UTIs), showed profound resistance to commonly prescribed oral agents. Resistance to the “Access” group antibiotic trimethoprim-sulfamethoxazole was 94.6% (Table 1). Furthermore, high rates of resistance were seen against “Watch” group agents, including ofloxacin (90.9%) and cefotaxime (85.4%) (Table 2). This leaves very few effective oral options for common community infections. The most effective agents against E. coli were “Watch” group carbapenems (meropenem, 12.6% resistance) and aminoglycosides (amikacin, 5% resistance), forcing a reliance on injectable, second-line therapies (Table 2).

*Pseudomonas aeruginosa* also displayed a multi-drug-resistant profile, with extremely high resistance to amoxicillin (98.4%) from the “Access” group (Table 1) and “Watch” group fluoroquinolones like ofloxacin (98.3%) and ciprofloxacin (87.4%) (Table 2). Alarmingly, ceftazidime, a key anti-pseudomonal “Reserve” agent, was ineffective against 19.6% of isolates (Table 3).

*Acinetobacter baumannii* emerged as a particularly formidable pathogen, with data showing 100% resistance to multiple “Watch” group antibiotics, including ceftriaxone, cefuroxime, and meropenem (Table 2). This indicates the circulation of extensively drug-resistant strains for which treatment options are severely limited. Similarly, Klebsiella species showed 100% resistance to the “Access” antibiotic ampicillin (Table 1), the “Watch” antibiotic cefotaxime (Table 2), and 87.5% resistance to ceftriaxone (Table 2).

## Discussion

This systematic review reveals a catastrophic landscape of AMR in Somalia. The findings show that resistance rates are not just high but are among the most critical reported globally, severely limiting viable treatment options for a wide range of common bacterial infections. The clinical and public health implications of this situation are profound, representing a critical failure of “Access” group antibiotics as defined by the WHO AWaRe framework and highlighting an urgent need for robust AMS efforts.

A key finding of this review is the near-universal prevalence of MRSA (97.4%). This Fig. 1 is dramatically higher than the 35-47% methicillin/oxacillin resistance reported in a comprehensive systematic review from neighboring Ethiopia [26,27]. This suggests that standard treatments for staphylococcal infections, such as those involving “Access” group beta-lactam antibiotics, are almost certainly obsolete in Somalia. The situation with Gram-negative pathogens is equally dire. The 94.6% resistance of *E. coli* to trimethoprim-sulfamethoxazole starkly contrasts with the 59% rate in Ethiopia [28]. For UTIs, this means one of the most common and affordable “Access” group first-line treatments is practically useless. Similarly, the 53.3% resistance to ciprofloxacin in Somalia far exceeds the 26% reported in Ethiopia, compromising the utility of fluoroquinolones, which are categorized as “Watch” antibiotics by the WHO and often considered second-line agents [29,30].

The emergence of extensively drug-resistant pathogens like *A. baumannii*, with 100% resistance to carbapenems, which are key antibiotics in the WHO Watch and Reserve categories, is a harbinger of a post-antibiotic era. These infections, typically acquired in healthcare settings, are exceptionally difficult to treat and are associated with high mortality rates [31]. This indicates severe deficiencies in hospital-based infection prevention and control. The absence of formally documented AMS programs to date further compounds this issue, highlighting the critical need for their urgent and structured implementation.

The drivers behind this hyperendemic resistance are deeply intertwined with Somalia's socio-political context [32]. The collapse of the formal health sector has led to an unregulated pharmaceutical market where antibiotics of often dubious quality are available without prescription [33]. Lack of access to diagnostic testing means nearly all prescribing is empirical, and without local data, this is tantamount to guesswork [34]. This creates immense and relentless selective pressure for bacteria to develop resistance [35]. These findings underscore that international and even regional treatment guidelines are dangerously unsuitable for use in Somalia. As highlighted by Jamil et al. (2025), many LMICs lack robust national guidelines, reinforcing the need to develop or adapt existing frameworks [36]. Instead of applying external guidelines wholesale, clinical decisions must be guided by adapted recommendations informed by local data, which is currently not being systematically collected.

## Conclusion

Somalia is facing a public health emergency fueled by extreme levels of AMR. Standard empirical therapies for common bacterial infections are no longer reliable, posing a grave risk to patient safety and threatening to reverse decades of progress in public health. The comparison with regional data suggests the situation in Somalia is exceptionally severe, necessitating a unique and robust response. Immediate action is critical. Somalia must establish national AMR surveillance to create evidence-based local treatment guidelines while simultaneously implementing AMS and regulating antibiotic sales to curb misuse and prevent the spread of untreatable infections.

### Limitations

The strengths of this review lie in its attempt to provide the first consolidated overview of AMR in Somalia, a data-poor region of critical public health concern. However, it is limited by the likely small number of primary studies available for inclusion, the potential heterogeneity in their laboratory methods, and a probable bias toward hospital-based, urban settings, which may not reflect the full picture in community and rural areas.

## Declaration of competing interest

The authors have no competing interests to declare.

## Appendix Supplementary materials

Image, application 1

Image, application 2
