# Supplementary material for: Prevalence of antimicrobial resistance in Somalia: A systematic review
Source: IJID Reg. 2025 Nov 4;17:100800. doi: 10.1016/j.ijregi.2025.100800 (PMC12704081; doi:10.1016/j.ijregi.2025.100800)
Supplement: Supplementary file 1 [file mmc1.docx]

Duplicate records were removed (n = 3).

Records identified through database searching (n = 22)

**Identification**

Records excluded

(n = 4)

(focused on TB)

Records screened

(n = 19)

**Screening**

Records excluded

(n = 3)

(Studies without quan­ta­ve AMR) data

Reports sought for retrieval

(n 15)

Studies included in review

(n =12)

**Included**

Fig. 1. Flow of information through the different phases of a systematic review
